# Quantifying the Evolution of Binder/Active Material Interface Fracture Properties from the As-Prepared State to Cycling Conditions

**DOI:** 10.1021/acsaem.5c02623

**Published:** 2025-10-21

**Authors:** Akshay S. Pakhare, Gordon H. Waller, Siva P. V. Nadimpalli

**Affiliations:** † Department of Mechanical Engineering, 3078 Michigan State University, East Lansing, Michigan 48824, United States; ‡ Chemistry Division, U.S. Naval Research Laboratory, Washington, District of Columbia 20375, United States

**Keywords:** interface stability, Li ion batteries, interface failure, binder/active material, solid electrolyte interphase

## Abstract

The binder-active material interface failure has been a critical issue in rechargeable batteries, especially for high-performance materials with large (∼300%) volume changes. This interface is very complex and undergoes various changes over the course of fabrication to cycling in service. Here, PVdF/Si was chosen as a model interface, and the interface failure behavior is quantified in terms of critical energy release rate *G*_c_. Further, the effect of electrolyte and the electrochemical cycling on *G*_c_ was quantified using an experimental method, which includes a Michelson interferometer-based optical setup coupled with a blister test in an electrochemical cell. The *G*_c_ of the as-prepared dry PVdF/Si interface is 0.55 ± 0.10 J m^–2^, but it decreased to 0.31 ± 0.10 J m^–2^ (i.e., a 40% reduction) upon introducing the electrolyte. The interface fracture property, *G*_c_, further reduces to 0.26 ± 0.03 J m^–2^ (additional 21% decrease) due to subsequent electrochemical cycling. It was also observed that the measured *G*_c_ is independent of sample geometry and is only a function of materials that make up the interface. The surface analysis (SEM and XPS) showed that the crack propagation in these samples occurred at the interface (i.e., neither in the PVdF nor in the Si substrate). Therefore, the reduction in *G*_c_ is attributed to the changes in the bonding environment at the interface due to electrolyte solvents and subsequent chemical changes that occur at the interface due to electrochemical cycling.

## Graphical abstract

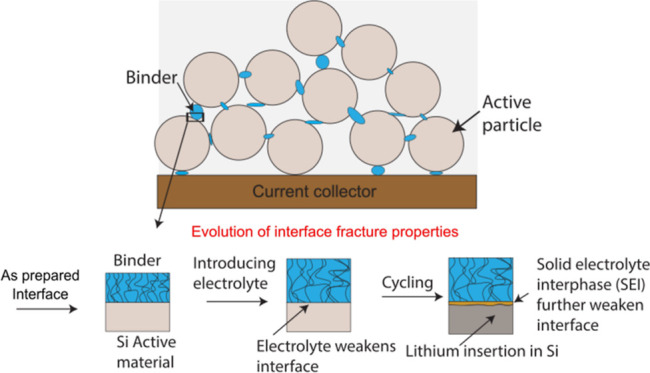

## 1 Introduction

Battery electrodes are porous composites consisting of active material (e.g., graphite, Si, and LiCoO_2_) particles, a polymer binder (e.g., polyvinylidene fluoride or PVdF and Na-Carboxyl methyl cellulose or CMC), and a conductive additive mix (as shown in Figure 1a). The mechanical integrity of the polymer and the polymer/active material interface is crucial for successful operation of a battery^1^ because the binder mix not only holds the active material particles together but also provides an electrical pathway to sustain electrochemical reactions at the active particle surfaces. The recent increase in the demand for high-capacity energy storage devices has shifted the focus to finding high-performance electrode alternatives to conventional graphite electrodes; materials such as Si (3579 mAh g^–1^),^2^ Al (1000 mAh g^–1^),^3^ and Sn (660 mAh g^–1^)^4^ are some promising options to replace graphite, whose specific capacity is only 372 mAh g^–1^. One of the main issues with the high-performance electrode materials is that they undergo large volume changes during electrochemical cycling. For instance, Si undergoes a ∼300% volume change upon reacting with Li;^5^ similarly, Al and Sn undergo ∼100%^6^ and ∼300%^7^ volume change, respectively, upon lithiation. This level of volume change (i.e., 100 to 300%) is known to cause fracture of active material particles,^8,9^ but more importantly, the binder which holds these active material particles together should be able to accommodate this level of deformation while maintaining mechanical integrity and the overall electrical network of the electrode. The binder/active material interface failure leading to electrical isolation of the active material particles in high-capacity electrodes with large volume expansion is one of the key mechanisms of capacity fade.^10^

**Figure 1:**
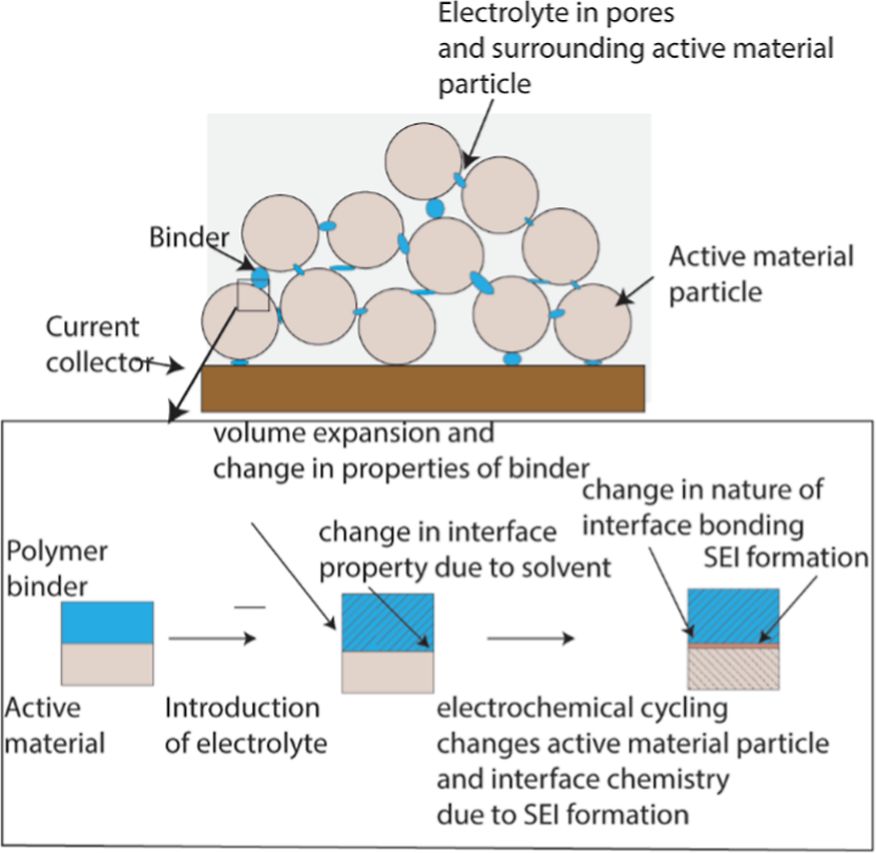
Various constituents of a commercial lithium-ion battery electrode. The inset shows how the binder/active material interface evolves from fabrication to the state after a few electrochemical cycles.

The conventional Li-ion positive electrodes (typically lithium-containing transition metal oxides with a layered crystal structure) and the negative electrode, mainly graphite,^11^ undergo a volume change of less than 10% compared to the ∼300% change of high-performance materials. The conventional binder materials such as polyvinylidene fluoride (PVdF) and Na-carboxyl methyl cellulose (CMC)+styrene butadiene (SBR) have been typically paired with these low-volume expansion materials, and these binders showed poor cyclic response when used with the high-performance materials, primarily due to polymer/active material interface failure.^10,12,13^ For example, studies on composite Si electrodes with PVdF binder showed significant loss of capacity within the first few cycles.^14^ Similarly, Sethuraman et al.^15^ observed that a Si-based composite electrode with a PVdF binder failed after only one cycle, which was attributed to the inability of PVdF to maintain interface integrity during large volume change of Si.

Since polymer binders play a key role in the electrode integrity, a large number of studies were focused on identifying new polymeric materials for binders: nature-inspired (alginates, xanthan gum, guar gum, and gum arabic),^16–18^ aqueous solvent-based (polyacrylic acid, chitosan, and brown algae),^19,20^ side chain-based (PEFM conductive polymer, PPyE: 1-pyrenemethyl methacrylate with triethylene oxide comonomer),^21,22^ and others (conductive polymer emulsion, lithium polyacrylate + alginate, polycaprolactone-based, and sodium methyl cellulose).^23–26^ The guiding principle in most of these studies has been to synthesize polymers with functional groups that promote strong bonding with active materials. However, these strategies are purely based on the qualitative observations and on composite electrode electrochemical cycling performance rather than the fundamental understanding of the binder/active material interface failure. In addition, the design approaches based on the bonding between polymer and fresh active material will not be sufficient, as the interface properties may evolve due to the introduction of electrolyte during cell assembly. In addition, the polymer-active material interface may evolve during cycling, further influencing the bonding behavior relative to an as-prepared, pristine binder.

Figure 1 shows how the polymer/active material interface conditions in the as-prepared dry electrode, which is the criteria used in aforementioned studies using composite electrodes, may change due to (1) solvent absorption by polymer when electrolyte is introduced;^27^ (2) phase change of active materials^5^ during the electrochemical reactions or change of solute concentration in active material during charge/discharge cycling; and (3) the formation of SEI products at the interface.^28^ The solvent absorption changes the mechanical behavior of the polymer and bonding environment at the active material/binder interface. Similarly, the phase changes and solvent concentration of the active material also affect the bonding at the interface. In a recent experimental study, Johnson et al.^28^ showed that SEI products indeed form at the interface of the PVdF/Si after cycling, which may also alter the bonding environment at the binder/active material interface. The binder/active material interface fracture property evolution occurs in both negative (anode) and positive (cathode) electrodes. Hence, it is important to understand the mechanics of binder/active interfaces to enable Si or other high-performance electrodes as a viable option and to provide the proper design criteria for polymer binders.

At present, there is no standard interface fracture characterization methodology for binder/active material interfaces. Although there are some initial attempts in this direction in terms of qualitative tests such as the peel test^29,30^ or scratch test^27,31^ on composite electrodes, these tests only provide an average qualitative property (i.e., an indirect indicator of polymer strength and the interface adhesion strength). Also, some of these strength experiments were performed under dry conditions, which does not account for the effect of electrolyte and the electrochemical conditions present inside a battery, which, as shown in Figure 1b, evolve over the course of fabrication and cycling. Recently, Pakhare and Nadimpalli^32,33^ characterized the fracture behavior of a PVdF/SiO_2_ interface in terms of critical energy release rate *G*_c_, which quantifies the ability of a material or interface system to resist crack growth, and they showed that this is a fundamental interface fracture property and is independent of geometry. The fracture mechanics approach with *G*_c_ as the failure criterion is very well established and successfully predicts failure in various interface systems such as polymer/metal interface,^34,35^ ceramic/polymer interface,^36,37^ and polymer/polymer interface.^38^ Theoretical studies on the fracture behavior of battery interfaces^39,40^ have been reported recently; however, a comprehensive experimental characterization of the polymer binder/active material interface is lacking.

Hence, the objective of this study was to propose an experimental method and characterize the interface failure of the binder/active material in terms of *G*_c_. Further, the aim is to understand and quantify how *G*_c_ of the binder/active material interface evolves from the as-prepared condition to the electrochemically cycled state. To this end, interface fracture samples that mimic the binder/active material interface were designed and fabricated using a series of micro- and nanofabrication methods. It should be noted that the proposed methodology is general and can be applied to any binder/active material interface system; here, the interface between PVdF and Si was chosen as a model interface. PVdF is typically used as a binder for cathodes in lithium-ion batteries; however, here it was chosen to characterize the fundamental interface fracture mechanics. The sample consists of a thin PVdF film on Si active material (or substrate). A novel electrochemical cell was designed to enable interface failure characterization not only under dry conditions but also under electrolyte soaking and electrochemical cycling. The interface fracture tests were performed by using a Michelson-based optical interferometer in conjunction with a thin film blister test setup. The tests were conducted on samples with a dry interface, after introducing electrolyte, and after electrochemical cycling to measure the evolution of the PVdF/Si interface property *G*_c_. The fracture surfaces were further characterized using scanning electron microscopy and X-ray photoelectron spectroscopy to elucidate the failure mechanism. The observations and data from this study will provide a methodology to understand interface failure in battery electrodes. Further, this will lay the foundation for capacity fade predictions in high-energy density electrodes due to binder/active material interface failure.

## 2 Experimental Methods

### 2.1 Sample Preparation

Figure 2a,b shows the fabrication process and schematic (section view) of the interface fracture sample, respectively. The process starts with cleaning of a single-side polished Si ⟨100⟩ wafer with organic solvents (acetone and isopropanol) to remove any surface contaminants, and then the Si wafer is dipped in Buffered Hydrofluoric Acid (BHF) to remove any native SiO_2_ on the surface. Next, a uniform solution of 10 wt % PVdF powder (Sigma-Aldrich product number 182702, average molecular weight of 534,000 by GPC) dissolved in NMP (1-Methyl-2-pyrrolidone) was applied onto the smooth side of the Si wafer by spin coating and subsequently vacuum-dried for 8 h at 110 °C. Thickness measurement using a profilometer resulted in a 1.2 μm thin film at 2500 rpm, whereas a rotational speed of 600 rpm produced a 6.8 μm PVdF thickness. The unpolished side of the Si wafer was coated with a Shipley Positive Resist (SPR 220, ∼5 μm thick) photoresist, which was selectively exposed to UV light using the Karl Suss MA/BA6 mask aligner using either a rectangular or circular mask pattern. The UV-exposed photoresist region was removed using an AZ 726 developer (MicroChemical, GmbH). This pattern of photoresist creates a rectangular or circular region where Si is exposed for removal using etching. The Si is first etched using Bosch process deep reactive ion etching (DRIE) (STS Pegasus 6, SPTS technologies) until ∼500 μm depth and further etching of Si (i.e., until a PVdF thin film is reached) is carried out using XeF_2_ etch (Xactix, SPTS Technologies). Since XeF_2_ gas does not react with PVdF,^41^ this two-step Si etching process avoids exposing PVdF to plasma and protects it. In the final step, a 50 to 100 nm Cu layer is deposited below the PVdF film to help in optical displacement measurements.

**Figure 2:**
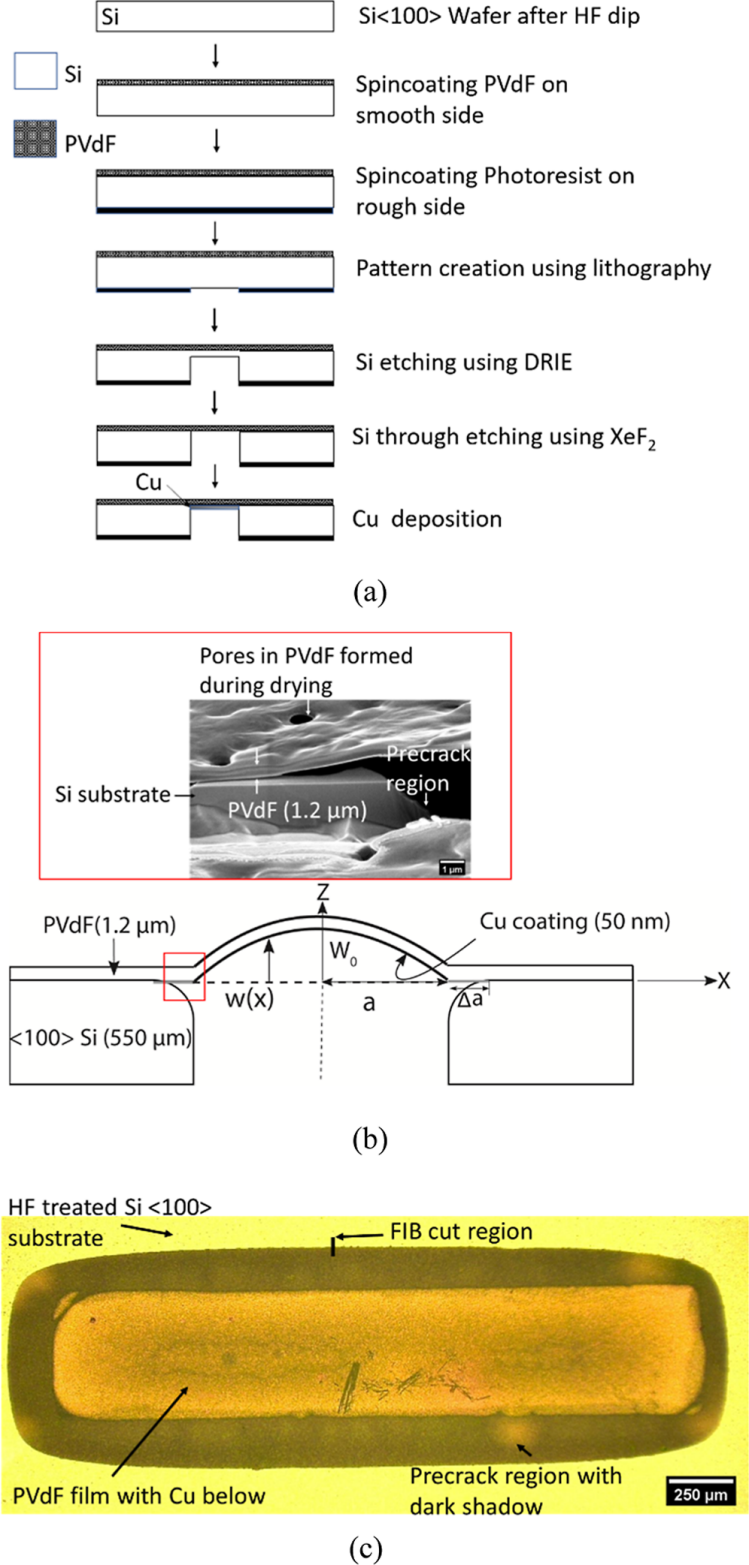
(a) Series of nanofabrication steps involved in the sample preparation; (b) section view of the PVdF/Si sample; and (c) micrograph of the top view of the rectangular plane strain sample. The dark band surrounding the rectangle is the precrack region (a FIB image from this region is shown in the inset of (b)).

Figure 2c shows a typical sample with the rectangular (850 μm width and 2800 μm length) free-standing region of the PVdF film; the circular (1390 μm diameter) freestanding PVdF sample dimensions are given in Figure S1. The sample shows two distinct regions: the bright reflective surface is the Cu beneath the PVdF film, and the dark shadow is a region below PVdF called the precrack region, which is shown as Δ*a* in Figure 2b. The inset of Figure 2b shows the image of the sectioned view of the precrack region obtained by a focused ion beam (FIB) cut; the details of the region, which includes a gap between the PVdF and Si substrate that acts as a precrack, can be seen in the figure. This precrack region was made possible by the two-step etching. The DRIE etch is anisotropic, i.e., preferred etching in the *z*-direction compared to the *x*-direction (ref. Figure 2a), which creates the vertical trench in Si, but the XeF_2_ etching is isotropic, i.e., similar in both *z* and *x* directions, creating the precrack region. The samples prepared here include the sharpest possible precrack from which the crack growth is initiated for *G*_c_ measurements, which is ideal as per the standard fracture test requirements.

### 2.2 Electrochemical Cell Assembly and Interface Fracture Measurement

Figure 3a shows the schematic of the electrochemical cell that was used to perform electrochemical lithiation of Si ⟨100⟩ through PVdF. The PVdF/Si sample is mounted in the cell using a chemical-resistant epoxy (LOCTITE STYCAST 2651 Henkel Adhesive). A polymer separator (triwoven fiber of polypropylene/polyethylene/polypropylene, from Celgard Inc.) soaked with electrolyte (1:1:1 wt % EC/DC/DMC with LiPF_6_ salt, Gotion Inc.) was placed between the Li foil and PVdF film of the sample. Here, the PVdF/Si sample acts as a working electrode, and Li foil acts as the counter and reference electrode. The circumferential edge of the Si wafer was covered with Loctite adhesive from HENKEL (Figure 3c) to prevent lithiation from the edges of Si and to enable Li flux only through the PVdF to ensure that the Si surface at the PVdF/Si interface gets lithiated, as shown in Figure 3e. A galvanostatic current density of 1.25 μA cm^–2^ was applied, and the cycling was carried out inside a glovebox (MBraun Inc., filled with Argon, <0.1 ppm O_2_ and <0.1 ppm H_2_O).

**Figure 3:**
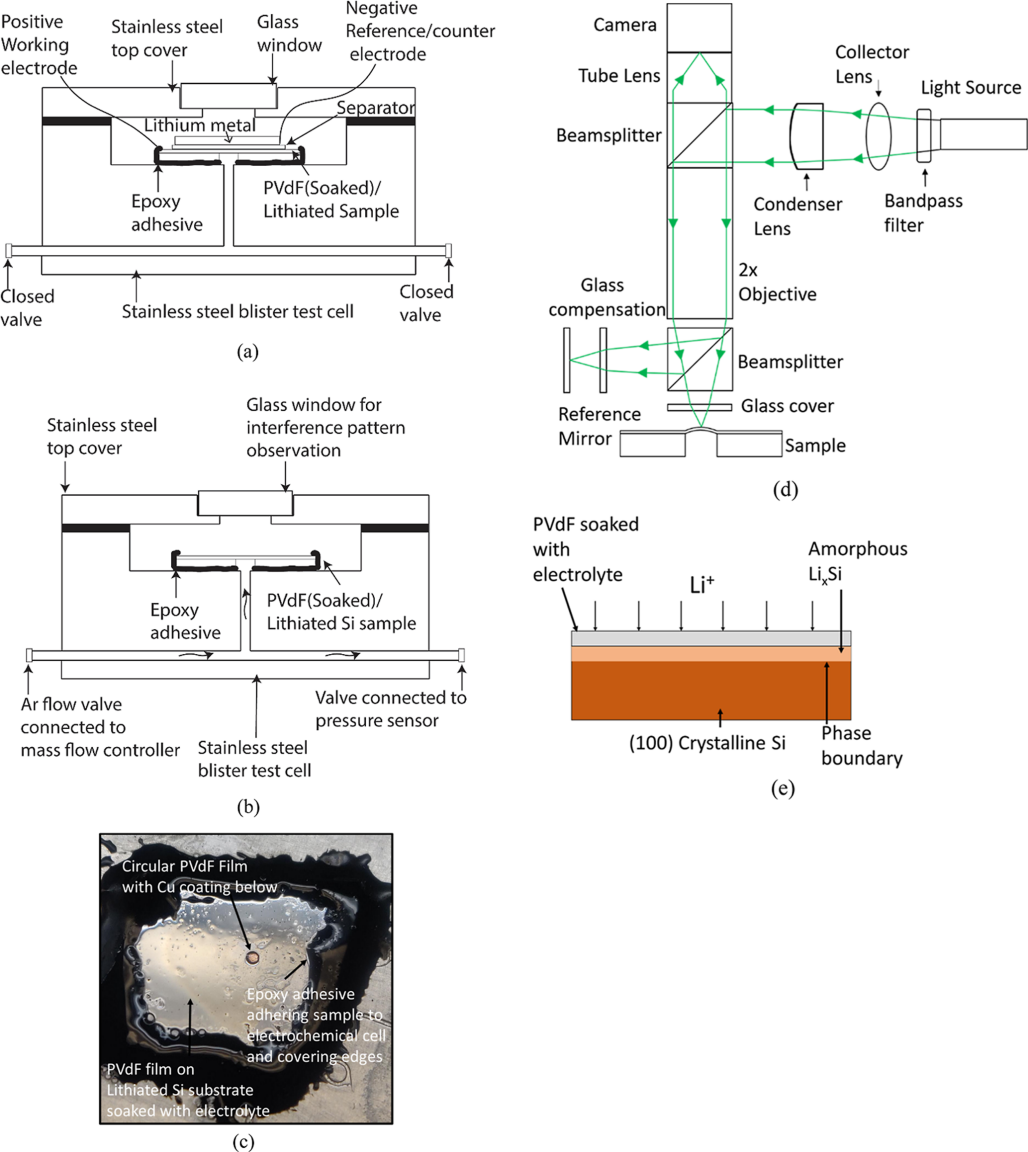
(a) Schematic of an electrochemical cell used to lithiate Si through the PVdF film. The sample prepared as described in Section 2.1 is bonded to a stainless steel beaker using epoxy. (b) Same setup after removing the Celgard separator and lithium foil to enable the fracture test (i.e., PVdF film pressurized with Ar to induce interface delamination). (c) Top view of the actual PVdF/Si sample after being subjected to lithiation. (d) Schematic of the experimental setup to measure the out-of-plane deflection (with ∼130 nm resolution) of the PVdF film using the Michelson interferometry principle. (e) Typical crystalline-to-amorphous phase transformation due to the reaction between Li and (100) Si and the phase boundary propagation, which consumes the Si substrate.

The interface failure tests were performed in the same cell, but as shown in Figure 3b, the separator and lithium foil were removed prior to the fracture experiment; this allows visual observation of the freestanding region of the PVdF film and the interface. The ultrahigh-purity Ar was used to pressurize the PVdF film through an orifice, as shown in Figure 3b. To capture the initial deformation of the film accurately, a slow mass flow of 0.2 sccm was used until 0.5 kPa because a small change in pressure produces large deflections during initial loading for blister test samples.^42^ After reaching 0.5 kPa, a constant mass flow rate of 0.8 sccm was maintained until the PVdF film delaminated from the Si substrate completely. The constant mass flowrate of 0.8 sccm resulted in a pressure increase of ∼0.5 kPa/min on the PVdF film during the test. The pressure (*p*) in the cell was measured using a pressure sensor (Omega PX309–050a5v) with a resolution of ∼0.1 kPa. The central deflection (*w*_0_) of PVdF under the applied pressure was measured using an in-house Michelson interferometer setup shown in Figure 3c.^32,33^ Images of the interference pattern on the sample were captured every 200 ms. A monochromatic (green) light with a wavelength of 514 ± 1 nm was used. A smaller bandwidth improves the coherence length of the light, which enhances the range of deflection measurement capability,^43^ and the ±1 nm bandwidth used here resulted in a spatial resolution of ∼5 μm and out-of-plane displacement resolution of ∼130 nm. Although photoelasticity and digital image correlation (DIC) methods have been used to characterize interface fracture behavior, there are limitations: (i) photoelasticity is limited to optically transparent birefringent materials, and (ii) DIC method may be challenging for samples submerged in liquids (e.g., electrolyte). The interferometry technique used here is versatile and general.

Besides the deformation of the PVdF film, the pressure corresponding to the onset of crack growth at the PVdF/lithiated Si interface, i.e., the failure pressure, is another critical measurement in determining the interface fracture property. The failure pressure was primarily determined by visual observation (from the recorded video and pressure measurements). This method worked very well in identifying the critical pressure in most of the samples, but the presence of electrolyte at the interface posed challenges in some of the cycled samples. Therefore, in addition to the visual method, a method based on sample compliance was used to identify the crack initiation pressure.

Compliance-based methods have been widely used in identifying crack initiation and propagation loads in standard fracture tests.^44^ Figure 4 shows the compliance method used here to identify the critical pressure. The solid blue curve shows a typical pressure-central deflection (*p-w*_0_) response of the PVdF/lithiated Si sample, and the dashed black curve shows the slope of this curve, i.e., *dw*_0_/*dP* or the sample compliance. When a membrane, such as the free-standing PVdF film in the samples here, deforms elastically and no other irreversible process (such as the crack growth in the PVdF/Si interface) occurs, the pressure-central deflection response is cubic in nature, i.e., *p*∝*w*_0_^3^, and the compliance is *dw*_0_/*dP* ∝ 1/*P* ^2/3^. As a result, the compliance *dw*_0_/*dP* continues to decrease with pressure and reaches a limit (i.e., approaches zero) when *p* becomes a very large value. Figure S2 in Supporting Information shows more details on this behavior. However, when there is crack initiation and growth, as in the PVdF/Si samples, the compliance *dw*_0_/*dP* starts to increase with *p* at the onset of crack initiation. Note from Figure 4 that after 3 kPa the compliance increases with pressure, which is an indication of crack initiation/growth. Here, a 15% increase in compliance was used to identify the crack initiation, indicated with a “x” symbol. The critical pressure from this method matches very well with the crack initiation pressure obtained with the visual observation (i.e., a pressure corresponding to a 50 μm crack) indicated with an “o” symbol in Figure 4.

**Figure 4:**
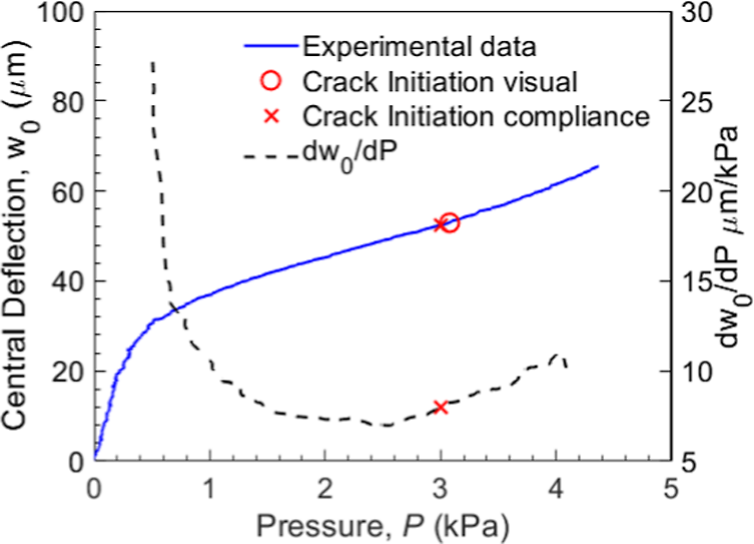
Typical pressure-central deflection (in solid blue) of the PVdF film in a plane strain sample along with sample compliance, i.e., the slope of the solid blue curve *dw*_0_/*dP* (in dashed black). The symbols “x” and “o” indicate the crack initiation event determined from the compliance method and the visual observation method, respectively.

### 2.3 Surface Chemical Composition Analysis Using X-ray Photoelectron Spectroscopy

In order to determine the PVdF/Si interface failure mechanism, XPS analysis was performed on the fracture surface (on the substrate, i.e., Si side), i.e., the Si surface was analyzed after completely delaminating the PVdF film from it using the methodology described in Section 2.2. The chemical analysis was performed using Kratos Axis ultra DLD with a monochromatic Al source. Charge correction was performed using the C 1s reference at 284.8 eV. The elemental composition and core scan deconvolutions were performed using CASA XPS software with Shirley background subtraction. The chemical compounds present at relative binding energy on the fracture surface were identified using the NIST XPS database.

### 2.4 Finite Element Method

Abaqus finite element software^45^ was used to simulate the fracture experiments of PVdF/Si and PVdF/Lithiated Si samples. The deformation behavior of pressurized films, such as the PVdF film, consists of small strain but large rotations;^32,46^ hence, the NLGEOM ON option in Abaqus that simulates the large deformation kinematics was prescribed. Figure 5 shows the finite element mesh of the fracture sample along with boundary conditions that are consistent with the experimental conditions. The thickness of the substrate does not affect the stresses either in the PVdF film or at the film/substrate interface; hence, it was adjusted to 100 μm to optimize computation time. Similarly, the stresses in the PVdF film, in the substrate, or at the interface, far from the crack tip in the radial distance, tend to be negligible (see Figure S3); hence, only a portion of the sample dimension was modeled.

**Figure 5:**
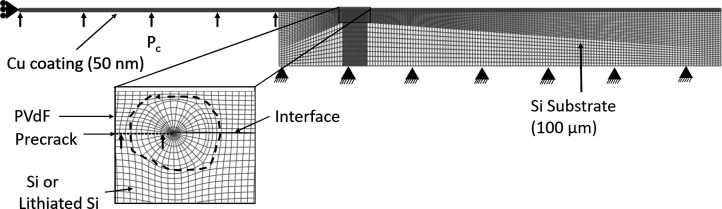
Finite element mesh of the PVdF/Si fracture sample along with the boundary conditions that are consistent with the experiments.

The PVdF film, substrate, and Cu coating were discretized with the eight-noded continuum plane strain quadrilateral elements CPE8R for rectangular samples and CAX8R for circular samples. The SEI layer does not contribute to the overall compliance of the system; hence, it is not included in the model. At least ten elements were included across the thickness of the PVdF film, with the smallest element size being 25 nm. To capture the stress singularity, the region around the crack tip, i.e., the first ring of elements surrounding the crack tip in the inset of Figure 5, is modeled using special 6-noded wedge elements called singularity elements. These singularity elements are formed by collapsing 3 of the 8 nodes of a CPE8R or CAX8R element. The mesh grading and the element sizes in Figure 5 were optimized through a mesh sensitivity study, and further reduction in element size did not produce any significant change in the results.

The mechanical behavior of the PVdF polymer (both dry and PVdF soaked under the electrolyte) was defined as a rate-dependent isotropic hardening model with the flow rule given as

$${\dot{\overset{®}{e}}}^{p}=D{\left(\frac{\mathrm{\sigma ̅}}{\sigma^{0}}-1\right)}^{n}$$ 2

where ${\dot{\overset{®}{e}}}^{p}$ is the equivalent plastic strain rate. The *D* and *n* are the reference plastic strain rate and stress exponent, respectively. The σ^0^ is the static yield stress, and $\mathrm{\sigma ̅}=\sqrt{\frac{3}{2}S:S}$ is the equivalent stress, where ***S*** is the deviatoric stress. The experimental true-stress and true-strain data of dry PVdF from Santimetaneedol et al.^47^ and soaked PVdF from Borges et al.^48^ was provided as input to the model. The Si and Cu are modeled as linear elastic materials. Crystalline Si(100) has a Young’s modulus of 160 GPa; however, when lithiated, it transforms into amorphous Si.^5,49^ The Young’s modulus of lithiated silicon in the amorphous phase is 43 GPa^50^ and remains nearly constant between Li_1.0_Si and Li_3.0_Si, which is used in the current simulation along with a Poisson ratio of 0.28. The material parameters are summarized in Table 1.

**Table 1:** Mechanical Properties of Materials Used in Finite Element Simulations

material	parameter	values	remarks
Cu	Young’s modulus	130.0 GPa	Freund and Suresh^53^
	Poisson ratio	0.34	
Si	Young’s modulus	160 GPa	Hopcroft and Nix^54^
	Poisson ratio	0.28	
lithiated Si	Young’s modulus	43 GPa	Sethuraman et al.^50^
	Poisson ratio	0.28	

The critical energy release rate (*G*_c_) of the PVdF/Lithiated Si interface was obtained by evaluating the contour integral (or J-integral) around the crack tip at the critical pressure; a typical contour is shown in the inset of Figure 5. As expected, the J-integral^51,52^ value evaluated for various contours (50) surrounding the crack tip was constant, i.e., it was path independent. Since the crack at the biomaterial interface undergoes mixed mode fracture, the phase angle ψ = tan^–1^(*G*_II_/*G*_I_), i.e., the ratio of shear to normal component of stress or energy release rate, was also evaluated. The phase angle (or mode-mixity) is evaluated using virtual crack closure technique. The energy release rate or *G* also depends on the residual stress in the film/substrate system (PVdF/Si or Lithiated Si substrate); the measured residual stresses of the samples were included in the simulations as initial conditions.

## 3 Results and Discussion

### 3.1 Electrochemical Cycling of the Interface Fracture Sample

Figure 6 shows the potential-time response of a PVdF/Si sample (Figure 2c) cycled under the galvanostatic process (with a constant current density of 1.25 μA cm^–2^) using the setup shown in Figure 3a. Note that the potential of the Si electrode drops sharply (to 0.6 V vs Li/Li^+^) from an open circuit potential (OCP) value of ∼2.7 V vs Li/Li^+^ (as an assembled electrochemical cell) at the beginning of the cycling, followed by a gradual decrease with time, reaching a stable plateau value of 0.115 V vs Li/Li^+^. The initial sharp drop is due to electrochemical polarization impedance, and the gradual decrease in potential is attributed to SEI formation. Obrovac et al.^5^ demonstrated with an X-ray diffraction study that the fully lithiated phase for silicon at room temperature is Li_3.75_Si, which was also supported in many reports, including the NMR studies by Song et al.^55^ and Trill et al.^56^ The constant potential (i.e., the plateau) observed in Figure 6 is an indication of the existence of a two-phase reaction, i.e., crystalline Si transforming into amorphous Li_3.75_Si as shown in the schematic (Figure 3e).^5,55^ The potential drops slightly below 0.115 V vs Li/Li^+^ before reaching a stable plateau to nucleate the amorphous lithiated silicon phase from crystalline (100) Si. Once the phase is nucleated, the phase boundary propagates at a steady pace, consuming the crystalline Si layer^49,57^ (as shown in the schematic of Figure 3e), resulting in a constant potential of 0.115 V vs Li/Li^+^. Here, the Si electrode was lithiated for 8 h to form a continuous lithiated Si layer underneath the PVdF film, and the constant potential shown in Figure 6 suggests that a uniform PVdF/lithiated Si interface exists in the interface fracture samples.

**Figure 6:**
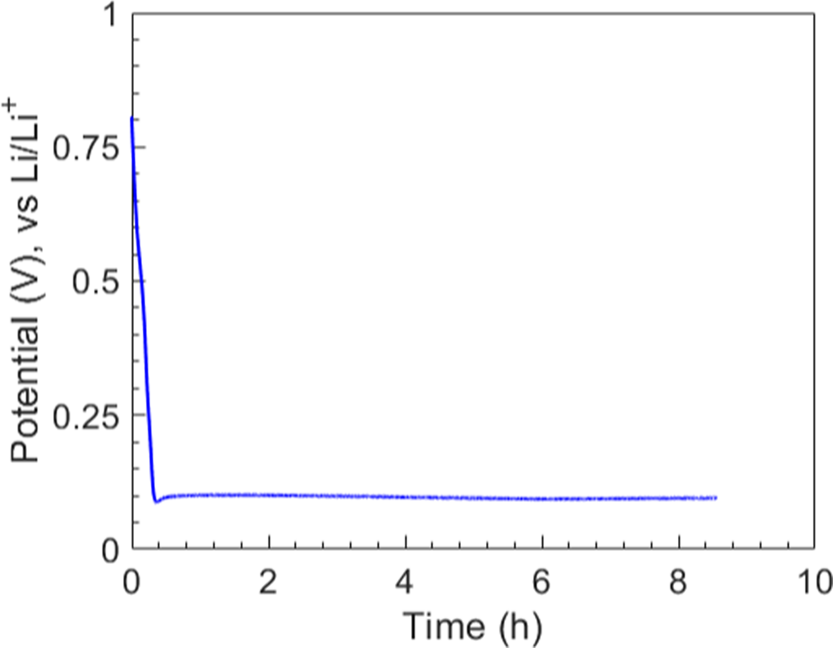
Potential vs time response of a PVdF/Si sample subjected to a galvanostatic lithiation reaction prior to fracture testing.

### 3.2 Mechanical and Fracture Behavior of the PVdF/Si Interface under Dry, Electrolyte Soaked, and Electrochemical Cycling Conditions

Figure 7a shows the central deflection-pressure response of the rectangular PVdF film in samples tested under dry (asterisk), soaked in electrolyte (dashed line), and electrochemically cycled (dashed–dotted line) conditions. A similar comparison is shown in Figure S4 for circular samples. As expected, the dry PVdF film shows a stiffer response, i.e., lower deflection for a given normalized pressure, because it not only has a higher modulus (1.6 GPa dry compared to 0.2 GPa^19,20,48,58^ under the wet condition) but also has a higher thickness (i.e., 6 μm compared to 1.2 μm in the other two samples). Since soft films, such as PVdF, on rigid substrates, such as Si, tend to wrinkle when subjected to compressive stresses (generated upon swelling due to solvent absorption), a relatively thinner, i.e., 1.2 μm thick PVdF film, was chosen for samples exposed to electrolyte (i.e., wet and electrochemically cycled) to minimize wrinkling. These thin free-standing PVdF films were fragile and posed several challenges in terms of handling; many samples were damaged in the fabrication, mounting, and testing phases. The data yield for the dry samples was higher (i.e., 90% success rate in measuring the *G*_c_) than the samples tested under the presence of electrolyte due to the challenges mentioned earlier and the increased compliance of already fragile films with the presence of electrolyte.

**Figure 7:**
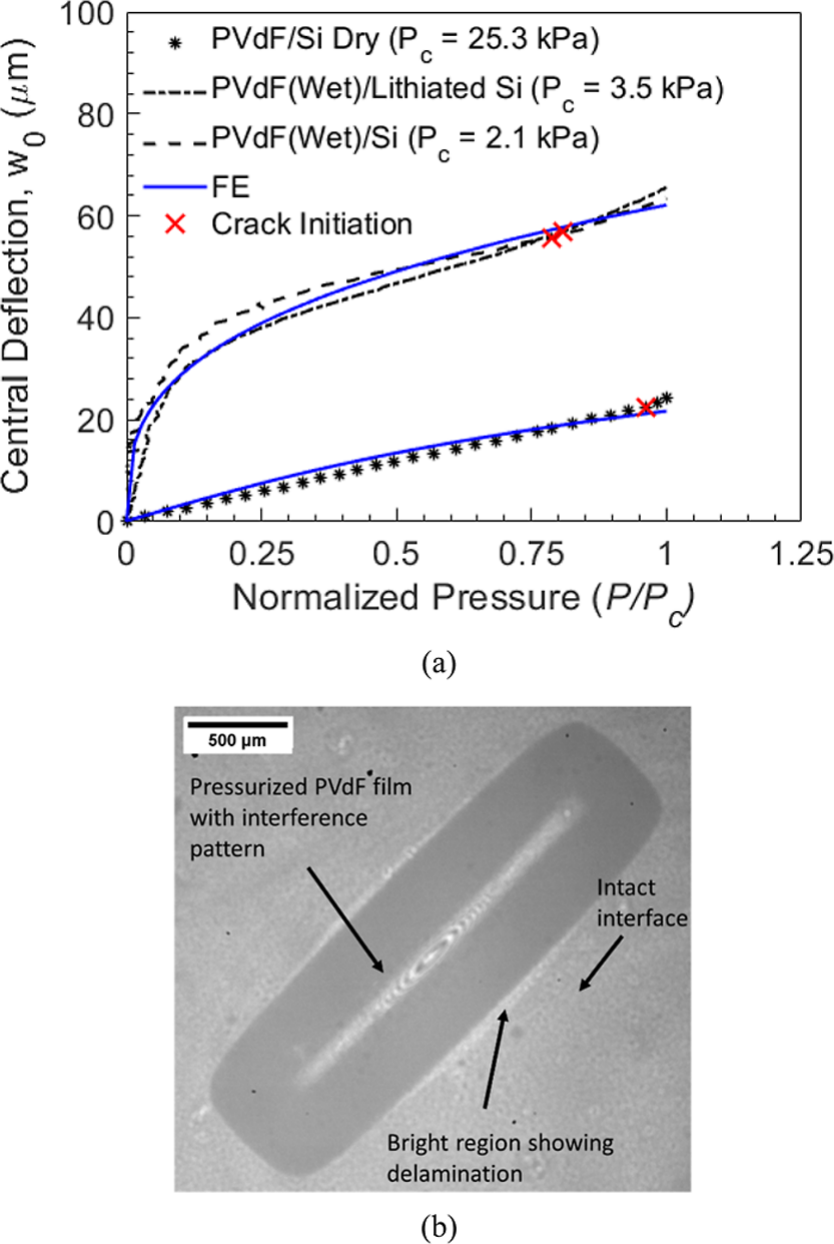
(a) Central deflection vs normalized pressure response of the PVdF film of dry or as-prepared samples (asterisk), PVdF­(wet)/Si (dashed line), and PVdF­(wet)/lithiated Si samples (dash-dotted line) along with the data from the corresponding FE simulations (solid blue line). Similar data from axisymmetric (or circular geometry) samples is presented in Figure S4. (b) Top view of sample with interference pattern on the PVdF surface, which provides the central deflection. The bright region at the edge of the sample is the interface crack or the delaminated zone.

Note from Figure 7a that the magnitude of central deflection of the film is significantly higher than the film thickness for all the samples, indicating that the PVdF film in these samples behaves more like a membrane, i.e., predominant stretching with negligible bending.^59^ Further, a good match between the experimental data and the FE results suggests that the prescribed boundary conditions along with the material data used in the simulations are accurate. The good match between FE and experiments in Figure 7a can also be attributed to the fact that appropriate initial conditions, i.e., the residual stress and the initial profile (Figure S5) of the free-standing PVdF film, were included in FE simulations.

The simulation data/curve deviates from the experimental curve at *P*/*P*_c_ ∼0.75 and 0.93 for wet and dry samples, respectively. This deviation in deflection behavior of PVdF is either due to the inelastic response^60^ of the PVdF membrane and/or due to interface failure, i.e., crack initiation and propagation.^61^ Since the inelastic behavior of the PVdF film was included in the FE model, the deviation observed is mainly due to crack growth. The initiation and propagation of a crack will affect the compliance and central deflection Δ*w*_0_ of the PVdF film and deviate from that of an intact PVdF film; the schematic in Figure S2 shows this behavior. The critical pressure identified with both the visual and compliance methods, indicated with “x” and “o” symbols, respectively, coincides with the deviation in Figure 7a, providing additional validation of the crack initiation pressure measurements.

Tables 2, 3, and 4 show the crack initiation pressure identified with two different methods: (i) compliance-based method and (ii) visual method in all the samples. As expected, the dry samples were able to sustain significantly higher pressure compared to the wet samples. One should exercise caution when comparing the critical pressures obtained from different samples, as critical pressure is not a fundamental property, and it depends on the material properties and geometry of the samples, as mentioned above. Note that for dry samples, the critical pressure obtained from both methods matched very well, with the visual method resulting in a slightly higher value. Similarly, the critical pressure from the visual method resulted in slightly higher values for the other two cases of samples (i.e., PVdF (wet)/Si and PVdF­(wet)/Lithiated Si). However, as mentioned earlier, the presence of electrolyte at the interface posed some challenges in identifying the crack initiation using the visual method in some cases, as noted in Tables 2–4.

**Table 2:** Crack Initiation Pressure (*P*_c_) for Dry Rectangular and Circular Samples Identified with the Compliance Method and Visual Observation^a^

	crack initiation pressure *P*_c_ kPa for dry PVdF/Si samples				
	rectangular		circular		
	compliance based	visual		compliance based	visual
sample 1	30.5	32.0	sample 1	26.2	28.4
sample 2	25.3	26.3	sample 2	25	26
sample 3	20.8	22.8	sample 3	33.8	34.9
sample 4	25.4	27.6	sample 4	23	23.8
			sample 5	33	34

a The PVdF film thickness in these samples was 6.8 μm.

**Table 3:** Crack Initiation Pressure (*P*_c_) for Wet PVdF/Si Rectangular and Circular Samples Identified with the Compliance Method and Visual Observation^a^

	crack initiation pressure *P_c_* kPa for PVdF(Wet)/Si samples				
	rectangular			circular	
	compliance based	visual		compliance based	visual
sample 1	5.0	7.7	sample 1	5.4	6.0
sample 2	2.1	2.6	sample 2	7.4	Difficult to observe
sample 3	4.5	7.0	sample 3	5.6	Difficult to observe

a The PVdF film thickness in these samples was 1.2 μm.

**Table 4:** Crack Initiation Pressure (*P*_c_) for PVdF­(Wet)/Lithiated Si Rectangular and Circular Samples Identified with the Compliance Method and Visual Observation^a^

	crack initiation pressure *P*_c_ kPa for PVdF(Wet)/Lithiated Si samples				
	rectangular		circular		
	compliance based	visual		compliance based	visual
sample 1	3.1	3.1	sample 1	4.0	4.0
sample 2	3.5	8.0			
sample 3	5.4	10.7			

a The PVdF film thickness in these samples was 1.2 μm.

The difference between the critical pressure obtained from the two methods is large for the wet samples compared to dry ones, which can be attributed to the sensitivity of the measurement method and challenges posed by the electrolyte at the interface. For example, the visual method relies on the difference in intensity/contrast created by a crack as shown in Figure 7b at the interface, which is very clear in the case of dry samples, but it was not clearly visible in some wet samples. In addition, the pixel size of the camera is ∼5 μm, and at least 10 pixels of data are needed for reliable identification of a 50 μm crack, but the deflection data is more sensitive (125 nm of displacement resolution); hence, the *P*_c_ from the visual method is slightly higher than the *P*_c_ obtained from the compliance method. The sample-to-sample variation in *P*_c_ was due to geometrical variation in (width of rectangular or radius of circular samples). The compliance method not only identified the critical pressure in all the samples tested here but also identified the critical pressure values that matched very well with that of the visual method; hence, the compliance method-based *P*_c_ from these experiments is used in determining the fracture properties.

### 3.3 Evolution of Interface Fracture Properties from the As-Prepared to Electrochemically Cycled Condition

Tables 5, 6, and 7 show the fracture property *G*_c_ of the PVdF/Si interface from the as-prepared dry sample, binder soaked with electrolyte, and electrochemically cycled samples, respectively, for both rectangular and circular sample geometries. Since *G*_c_ is a fundamental property that quantifies the ability of a material or interface to resist crack initiation or crack growth, it should be independent of sample geometry. Note from Table 5 that the *G*_c_ (average ± standard deviation) of the as-prepared PVdF/Si interface from the rectangular and circular samples is 0.61 ± 0.08 J m^–2^ and 0.49 ± 0.10 J m^–2^, respectively, and as expected, there is no statistically significant difference between these means (at 95% confidence interval, *t*-test), supporting the argument that *G*_c_ is a fundamental property. Further, the *G*_c_ values of the PVdF (wet)/Si interface presented in Table 6 for rectangular and circular geometries, 0.27 ± 0.1 J m^–2^ and 0.37 ± 0.09 J m^–2^, also show no statistically significant difference (with 95% confidence interval, *t*-test), further establishing the fact that *G*_c_ is independent of geometry. The *G*_c_ is a function of phase angle ψ (i.e., mode-mixity parameter); the phase angle for the dry PVdF/Si interface is 68°, whereas that for the PVdF­(wet)/Si and for the PVdF­(wet)/Lithiated Si interface is 65°. Since each table presents the data for a particular state of the interface (i.e., dry or as-prepared PVdF/Si, PVdF­(wet)/Si, and PVdF (wet)/Li_3.75_Si), the average of the samples from each table is presented in Figure 8a to show how *G*_c_ evolves as a function of the state of the interface. The reported adhesion energy values from peel tests^58^ and peak force values from scratch tests^27,31^ in the literature are geometry-dependent parameters and do not provide predictive capabilities. In contrast, the *G*_c_ obtained here is quantitative and geometry independent and provides predictive capability.

**Table 5:** Energy Release Rate (*G*_c_) of the Dry (As-Prepared) PVdF/Si Interface

	energy release rate *G*_c_ (J m^–2^) of the dry PVdF/Si interface		
	rectangular	circular	
sample 1	0.66	sample 1	0.58
sample 2	0.58	sample 2	0.53
sample 3	0.51	sample 3	0.55
sample 4	0.68	sample 4	0.34
		sample 5	0.47
mean and standard deviation	0.60 ± 0.07	mean and standard deviation	0.49 ± 0.09

**Table 6:** Energy Release Rate (*G*_c_) of the PVdF­(Wet)/Si Interface

	energy release rate *G*_c_ (J m^–2^) of PVdF(wet)/Si interface		
	rectangular	circular	
sample 1	0.33	sample 1	0.31
sample 2	0.15	sample 2	0.47
sample 3	0.33	sample 3	0.32
mean and standard deviation	0.27 ± 0.1		0.36 ± 0.09

**Table 7:** Energy Release Rate (*G*_c_) of the PVdF­(Wet)/Lithiated Si Interface

	energy release rate *G*_c_ (J m^–2^) of the PVdF(wet)/Lithiated Si interface		
	rectangular	circular	
sample 1	0.23	sample 1	0.20
sample 2	0.26		
sample 3	0.30		
mean and standard deviation	0.26 ± 0.03		

**Figure 8:**
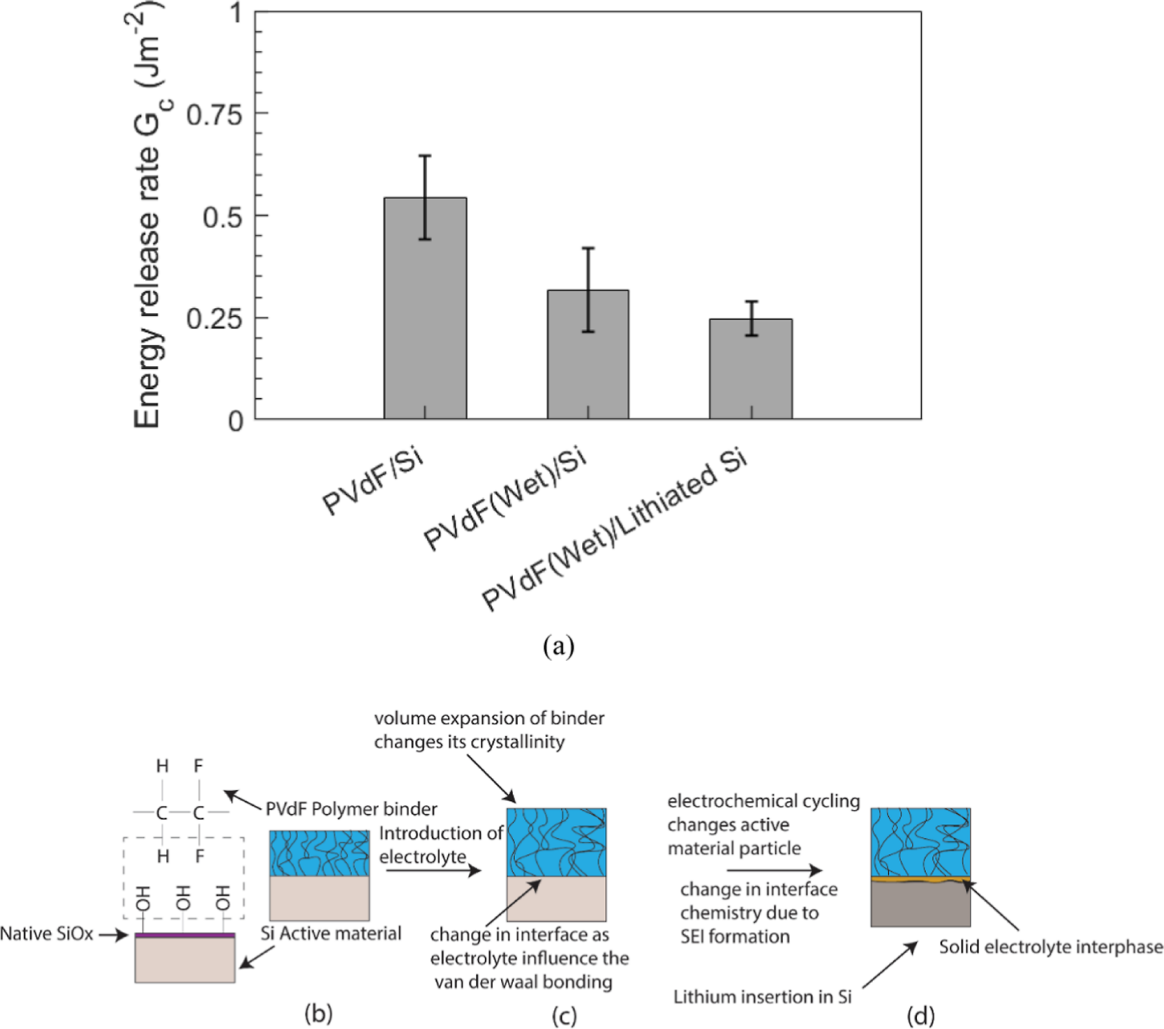
(a) Evolution of energy release rate *G*_c_ of the PVdF/Si interface from the as-prepared state to the electrochemically cycled state. The *G*_c_ of the interface decreased significantly (i.e., 40% decrease) when electrolyte was introduced, and it decreased further (20%) after electrochemical cycling. Schematic (b–d) shows changes that occur to the PVdF/Si interface, which is attributed to the *G*_c_ evolution shown in (a). (b) As-prepared interface, (c) changes to binder crystallinity due to electrolyte wetting, and (d) lithiation of Si and SEI formation at the interface.

Note from Figure 8a that the average *G*_c_ (from both rectangular and circular samples) of the as-prepared or dry PVdF/Si interface is 0.54 ± 0.1 J m^–2^, and it decreased to 0.32 ± 0.1 J m^–2^, i.e., nearly a 40% drop, due to the introduction of electrolyte; the interface property or fracture energy *G*_c_ further decreased to 0.25 ± 0.04 J m^–2^, i.e., a further 21% decrease, when the samples are cycled electrochemically. It is clear from the data that the *G*_c_ of the binder/active material interface is not constant and evolves under various conditions. Further, the data from Figure 8a suggest that the electrolyte plays a relatively more important role in influencing the binder/active material interface properties compared to the subsequent chemical changes at the interface due to electrochemical cycling. It is known that PVdF adheres to the Si surface via van der Waals forces of attraction,^1,19^ and the adhesion is likely to occur between the polymer chain and the –OH molecule on the Si surface (as shown in the schematic of Figure 8b). Also, the PVdF binder is known to absorb the EC, DEC, and DMC solvents.^58^ Hence, when the electrolyte is introduced, PVdF swells by absorbing the solvents in the electrolyte, and the solvents reduce the crystallinity of PVdF, which can reduce the critical loads sustained by the interface (Figure 8c).^27^ Further, when the solvents reach the PVdF/Si interface, they can influence the chemistry at the PVdF/Si interface, i.e., –OH bond (that contributes to the van der Waals force of attraction between PVdF and Si) can be affected by the solvent molecule due to the polarity associated with EC, DEC, and DMC.^62^ The solvent also leads to plasticization of PVdF, i.e., reduction in its yield strength and modulus due to reduction in its crystallinity,^27^ i.e., allowing polymer chains to slide under deformation easily. Evan et al.^63^ suggest that plasticization tends to increase fracture toughness by promoting blunting; however, chemical effects such as those caused by impurities, segregates, or moisture reduce toughness. The introduction of EC/DEC/DMC electrolyte solvents to the PVdF/Si interface has contributed to a 40% reduction in *G*_c_, as shown in Figure 8a.

Since the electrolyte used here was composed of EC/DEC/DMC solvents, it is possible that a different solvent chemistry could result in different interface fracture property values for the interface. It is also known that the SEI products formed at the interface and the absorption behavior of the binder depend on the type of solvents used in the electrolyte.^64^ The van der Waals interaction, or chemical bonding, at the interface changes further under electrochemical cycling due to the formation of SEI products at the interface^28^ and the insertion of Li in the Si matrix (Figure 8d). A detailed XPS analysis of the fracture surfaces from the PVdF/Lithiated Si interface samples did indicate the presence of SEI products at the interface. These interface SEI products, details of which are presented in the next section, and the reaction between Si and Li, which changes the interface chemistry between PVdF and the lithiated Si surface, resulted in further reduction of *G*_c_ to 0.25 ± 0.04 J m^–2^, as shown in Figure 8a.

One of the requirements of *G*_c_ measurements is the existence of small-scale-yielding condition at the crack tip in the fracture samples. In other words, the mechanical behavior of the sample must be nominally elastic prior to fracture with the exception of a small region near the crack tip, where nonlinear processes such as plastic dissipation and bond breaking occur. Figure 9a–c shows the equivalent plastic strain contours of the dry PVdF/Si, PVdF­(wet)/Si, and PVdF­(wet)/lithiated Si samples at the onset of crack growth. The blue region of the sample indicates elastically deformed regions with the color contours, especially the red color, showing plastic zones, and it can be observed that the plastic strain zones are significantly smaller than the thickness of the PVdF film, i.e., the size of the plastic zone is <10% of the film thickness. This represents small-scale yielding condition in fracture mechanics.^52,65^ This confirms that the *G*_c_ data presented here (in Tables 5–7 and Figure 8a) are the critical energy release rate or intrinsic interface fracture property.

**Figure 9:**
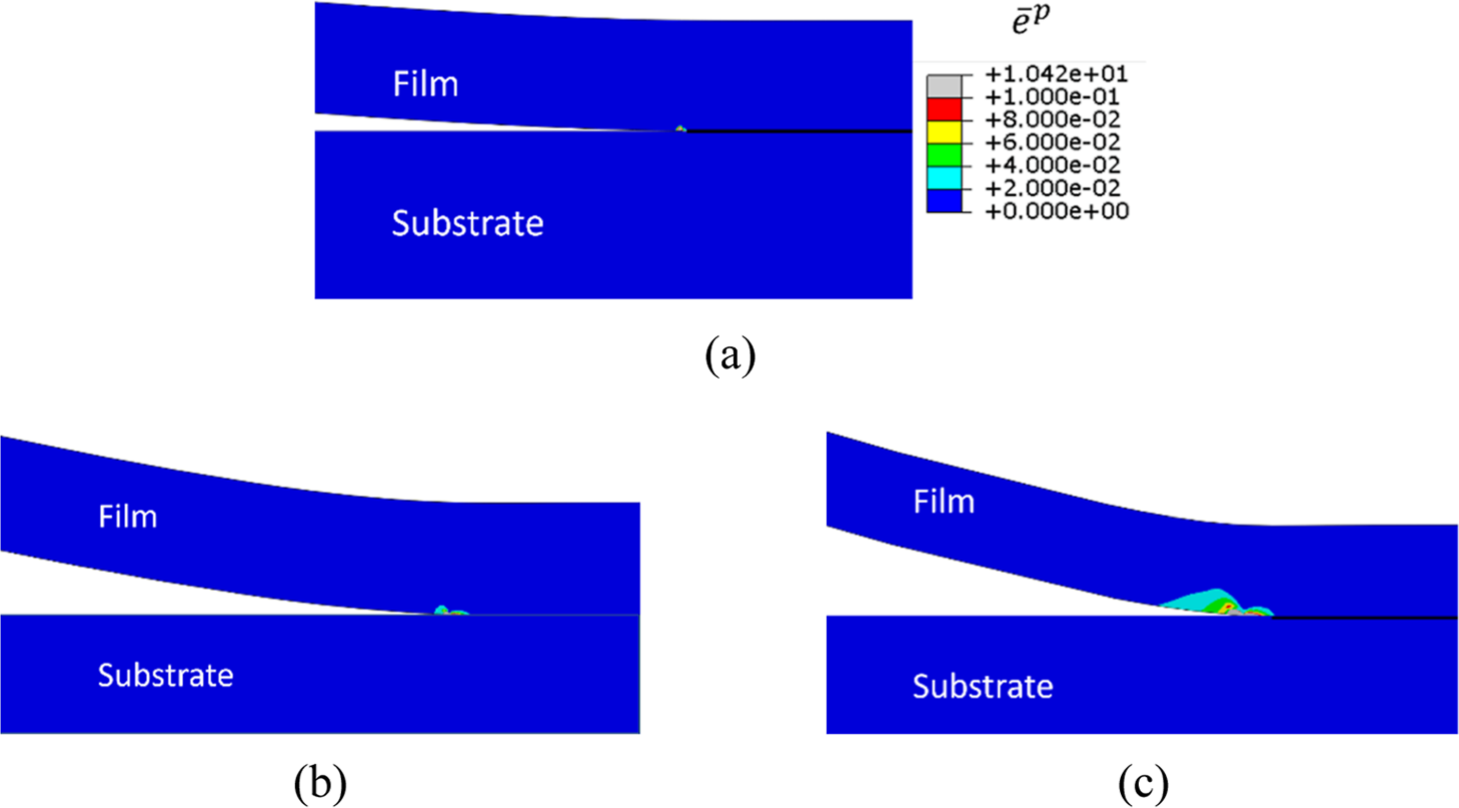
Equivalent plastic strain contours of (a) dry PVdF/Si, (b) PVdF­(wet)/Si, and (c) PVdF­(Wet)/Lithiated Si sample at the onset of crack initiation. The plastic zone, indicated with red color contour, is relatively small compared to film thickness, ensuring small-scale yielding conditions for *G*_c_ measurement.

Finally, the presence of an electrolyte at the interface could contribute to the mechanical forces due to its surface tension property. Hence, it is instructive to see what portion of the interface *G*_c_ is due to the contribution from the surface tension (γ) of the electrolyte. Assuming that the surface tension of the electrolyte used (1:1:1 wt % mixture of EC/DC/DMC) is similar to EC/DMC, which is 40.13 ± 0.20 mN/m,^66^ a simple force balance shows that it contributes ∼1.28 kPa of pressure ( $P_{\gamma }=\frac{C\gamma }{A}$ , where *C* is the perimeter of the rectangle and *A* is the surface area of free-standing PVdF). This estimate is very close to the experimental measurement; i.e., the pressure sustained when already failed samples were retested was close to ∼1.0 kPa, which is purely from electrolyte surface tension. This pressure is equivalent to 0.06 J m^–2^ of *G*_c_, which is ∼19% of the measured *G*_c_ of the wet interface.

### 3.4 Fracture Surface Analysis and Implications for *G*_c_

#### 3.4.1 Mechanical Process (or Crack Growth Mechanism)

Figure 10 shows three possible crack paths in the samples: (i) crack propagation in the PVdF film but near the interface, (ii) crack propagation in the Si substrate but near the interface, or (iii) crack growth at the interface, and each of these crack growth mechanisms results in a characteristic fracture energy value. For example, mechanism (i), i.e., crack growth in PVdF, would have resulted in a fracture energy close to ∼43 kJ m^–2^ ^67^ and mechanism (ii), i.e., crack growth in Si, would have resulted in fracture energy between 5 and 11 J m^–2^,^68^ but the measured *G*_c_ (Table 5 and Figure 8) is lower than these two. Hence, the *G*_c_ data, which is an order of magnitude less than these energy values, indicate that mechanism (iii) is the likely scenario, i.e., PVdF separated completely from Si (i.e., crack growth occurred exactly at the interface). To confirm this hypothesis, XPS analysis on the fracture surface of both dry and lithiated samples (on Si side), bare PVdF, and a pure Si surface was conducted, and the data are shown in Table S1. Since PVdF is the only source of F, the negligible concentration of F on the PVdF/Si fracture surface, which is similar to that of the pure Si surface, suggests that the failure occurred at the interface (i.e., mechanism (iii) polymer completely separated from the Si surface). The XPS analysis also shows that the dry PVdF/Si fracture surface is chemically different from the PVdF/Lithiated Si surface shown in Figure S6. Mainly the dry fracture sample represents surface chemistry as that of the Si wafer surface, whereas the lithiated Si surface has chemically transformed to a surface with SEI formation. It should also be noted that the Si used is atomically flat; therefore, any contribution due to mechanical interlocking can be neglected. The fact that the *G*_c_ value is low for the PVdF­(wet)/Si and PVdF­(wet)/Lithiated Si interfaces (Figure 8) compared to the dry interface indicates that the failure mechanism (iii) is the crack growth mechanism in these samples as well.

**Figure 10:**
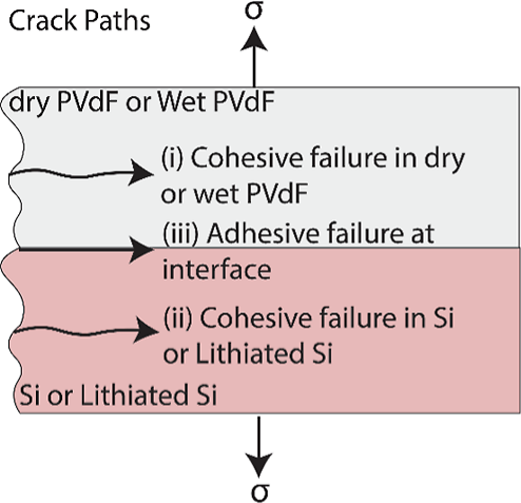
Schematic of possible crack paths that exist in the PVdF/Si fracture samples, each of which leads to a characteristic energy value.

#### 3.4.2 XPS Analysis of the PVdF/Lithiated Si Fracture Surface

As shown in Figure 8 the fracture resistance *G*_c_ of the as-prepared or dry PVdF/Si interface decreased significantly (by 40%) when electrolyte was introduced, and it was further decreased (another 21%) after electrochemical cycling. This 21% decrease was attributed to the change of bonding environment (i.e., Si to LixSi after cycling) at the interface; Johnson et al.^28^ showed that SEI products could form at the interface. Hence, to confirm whether the SEI products formed at the interface in the current samples, XPS analysis was carried out.

Figure 11 shows the XPS core scans of a typical fracture surface of the PVdF­(wet)/Lithiated Si sample for the Li, Si, P, C, O, and F elements. The deconvolution spectra of the Li region, in Figure 11a suggest that the red peak is LiF/Li_x_PF_y_O_z_/Li_x_PF_y_ formation.^69^ The Si core scan in Figure 11b shows peaks corresponding to SiO_2_ and the SiO_x_ surface. The possibility of not seeing a lithiation peak such as Li_x_Si_y_, as shown by Johnson et al.,^28^ could be due to the lithiation phase front thickness being low. Here, a total of 8 h of lithiation was carried out compared to 25 h of lithiation for multiple cycles; also, Johnson et al.^28^ performed through etching of the SEI film for depth profiling and to reach the lithiated Si surface compared to only surface analysis performed in this study. Figure 11c shows that P from Li-salt (LiPF_6_) and the PF_6_ solvated ions react with solvents to form SEI products such as Li_x_PF_y_ and Li_x_PF_y_O_z_.^28,70^ The Li_x_PF_y_O_z_ product is also seen in the F1s deconvolutions in Figure 11f. The C–O and Si–O in Figure 11e could have been formed when transferring the sample from the experiment to the analysis chamber. Table 8 summarizes the compounds and compositions of the PVdF/Si interface. This analysis clearly suggests that typical SEI compounds are found at the PVdF/lithiated Si interface and play an important role in decreasing the *G*_c_ value of the PVdF/Si interface. The *G*_c_ measurement presented here is extremely useful, as it can act as a design parameter to obtain durable interfaces in batteries by (i) choosing the right binder, (ii) modifying the binder and the interface using artificial SEI’s, (iii) designing microstructures which can mitigate the interface traction loading which causes failure, and (iv) the measurement method is not limited to the binder/active material interface but can also be performed for the other interfaces in batteries.

**Figure 11:**
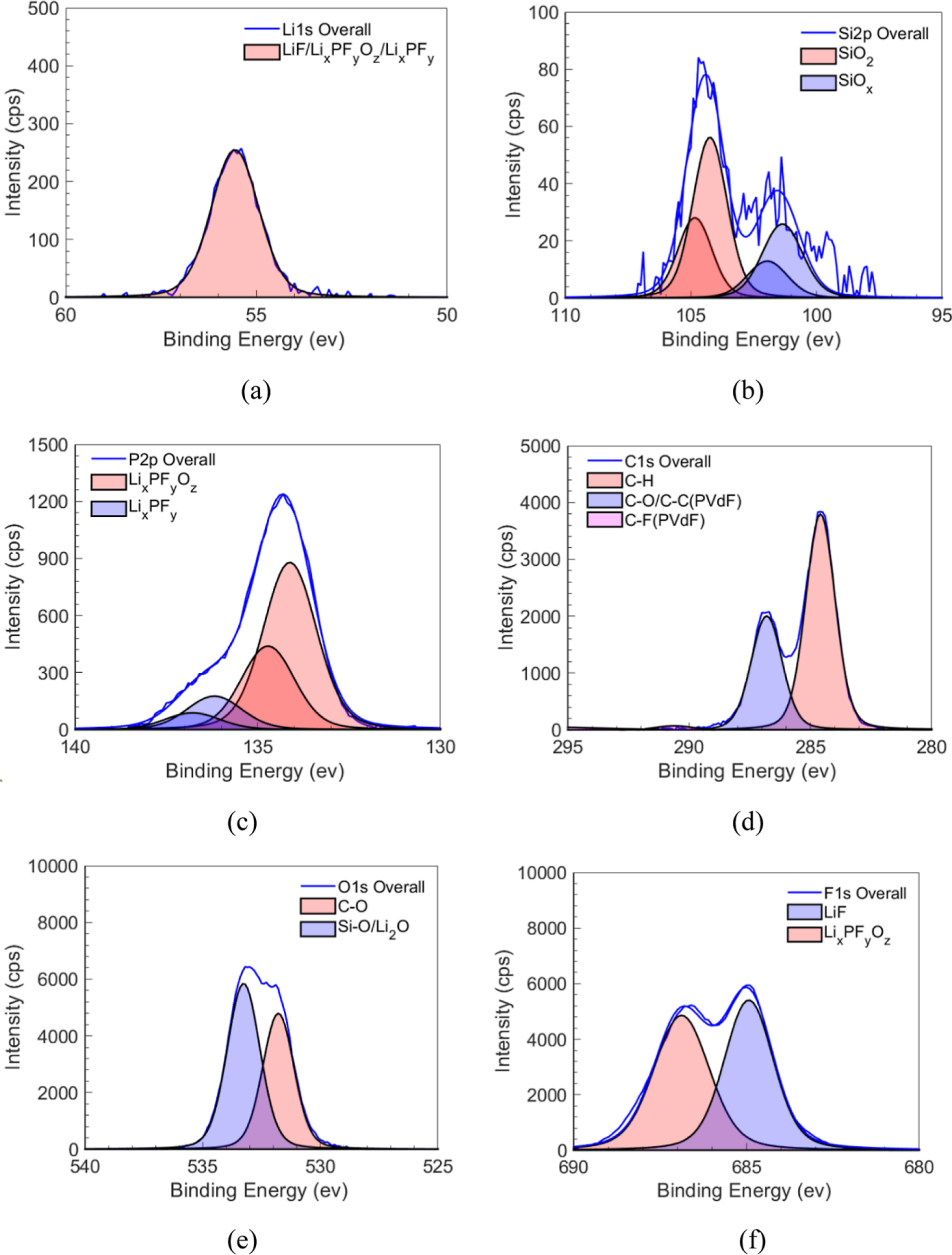
(a–f) Core scans of the fracture surface of PVdF­(Wet)/Lithiated Si performed at the peak of (a) Li1s, (b) Si2p, (c) P2p, (d) C1s, (e) O1s, and (f) F1s. The deconvolution was performed using CASA XPS software, and the peaks were identified referring to the NIST database. These peaks indicate the formation of SEI at the PVdF/Si interface.

**Table 8:** Assigned Peak to the Various Core Scan on the Fracture Surface along with the Binder Energy, Full Width at Half Maximum of the Peak, and Relative Composition for Each Core Scan

peak assignment	binding energy (eV)	full width half maxima (fwhm)	% composition at each core scan	atomic %
Li1s				
LiF/Li_*x*_PF_*y*_Oz/Li_*x*_PF_*y*_	55.56	1.47	100	38.22
Si 2p3/2				
SiO_x_	101.96	1.61	65.07	0.45
SiO_2_	104.84	1.58	34.93	0.24
P 2p3/2				
Li_x_PF_y_O_z_	134.29	1.72	82.70	5.45
Li_x_PF_y_	136.21	1.78	17.30	1.14
C1s				
C–H	284.59	1.44	63.30	18.74
C–O/C–C (PVdF)	286.81	1.50	34.96	10.34
C–F (PVdF)	290.60	1.90	1.74	0.52
O1s				
Si–O/Li_2_O	531.79	1.57	43.89	6.85
C–O	533.26	1.65	56.11	8.75
F1s				
LiF	684.93	1.90	49.50	4.61
Li_x_PF_y_O_z_	686.88	1.67	50.50	4.70

## 4 Conclusion

A novel experimental method is used to characterize the polymer binder/active material interface fracture behavior in terms of the critical energy release rate *G*_c_, and the effect of electrolyte and subsequent electrochemical cycling on the interface fracture property was quantified. Here, a PVdF/Si interface is chosen as a model interface. A blister test sample with the PVdF/Si interface was designed and developed to characterize the interface failure behavior. An electrochemical cell was designed to carry out electrochemical cycling followed by an interface fracture test without disturbing the sample. The pressure and deformation of the sample are recorded with a pressure sensor and an optical setup based on the Michelson interferometer, respectively. A finite element analysis of the experiment was carried out where the initial conditions, boundary conditions, appropriate constitutive behavior, and material parameters were included in the simulation. The FE results matched with the experimental pressure-deflection data. The critical pressure corresponding to the onset of crack growth in each sample was measured and was used in evaluating *G*_c_ (which indicates the ability of a material or interface to resist crack growth). As expected, the *G*_c_ is independent of sample geometry and is only a function of the interface system, i.e., the materials that makeup the interface. The *G*_c_ of the as-prepared dry PVdF/Si interface is 0.54 ± 0.1 J m^–2^. When electrolyte is introduced, i.e., the sample was exposed to electrolyte, the *G*_c_ of the PVdF/Si interface drops by 40% to 0.32 ± 0.1 J m^–2^. Further, when the sample is subjected to subsequent electrochemical cycling, the *G*_c_ further reduced to 0.25 ± 0.04 J m^–2^ (i.e., an additional 21% decrease from the wet case). The surface analysis (SEM and XPS) shows that the crack propagation in these samples occurred at the interface (i.e., neither in the PVdF nor in the Si substrate). Therefore, the reduction in *G*_c_ occurs through chemical changes at the interface, i.e., the changes that occur in the bonding environment at the interface. For example, the introducing of electrolyte causes PVdF to swell, and the solvent reaches the interface; the polar solvents in the electrolyte affect the bonding and influence interface adhesion. Further, when electrochemical cycling is performed, an SEI layer forms at the interface, which was verified through XPS analysis, and the reaction between Li and the active material (Si in this case) further changes bonding at the interface. This further reduces the interface energy.

It is clear from this study that the binder/active material interface strength does not remain constant and changes significantly. In this particular case of the PVdF/Si interface, the most significant (i.e., a 40%) reduction in interface strength (or resistance to crack growth) occurred due to the electrolyte and a further 20% reduction due to electrochemical cycling. The fracture mechanics framework presented here is more general and applicable to other binder/active material interfaces, solid–state interfaces, and cathode binder/oxide interfaces. Also, the *G*_c_ parameter presented here is a fundamental (geometry-independent) property that characterizes and quantifies interface fracture behavior as opposed to, for example, peel force/strength, which is a geometry-dependent qualitative parameter. Hence, the *G*_c_ measured here enables the failure prediction of electrodes. This lays the foundation for the degradation modeling of battery electrodes, which will be a valuable tool for designing durable batteries.

## Supplementary Material



## Notes

Details of circular sample geometry and testing; crack identification method; FEA mesh sensitivity study; and additional XPS analysis and data (PDF)
