# Supplementary material for: Quantifying the Evolution of Binder/Active Material Interface Fracture Properties from the As-Prepared State to Cycling Conditions
Source: ACS Appl Energy Mater. 2025 Oct 21;8(21):16122–35. doi: 10.1021/acsaem.5c02623 (PMC12606567; doi:10.1021/acsaem.5c02623)
Supplement: Supplementary file 1 [file ae5c02623_si_001.pdf]

Supporting Information

Quantifying the evolution of binder/active material interface fracture properties from as prepared state to cycling conditions

Akshay S. Pakhare[1], Gordon Waller[2], and Siva P.V. Nadimpalli[1, *]

[1]Department of Mechanical Engineering, Michigan State University, East Lansing, MI, 48824, USA

[2]Chemistry Division, U.S. Naval Research Laboratory, Washington, D.C., 20375, USA

[*]Corresponding author: E-mail: sivan@msu.edu

Tel: +1 517 432 2976, Fax: +1 517 353 1750

1. Top view of circular sample for interface fracture measurement

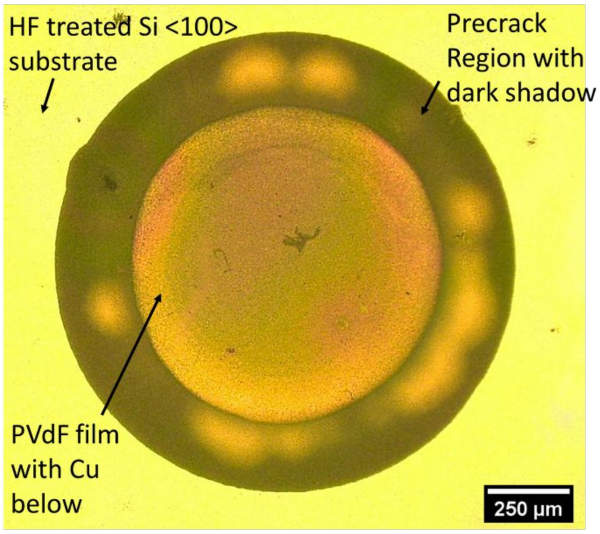

Figure S1 The top view of the circular sample (~1390 μm diameter). The dark band surrounding the circle is the pre-crack region similar to rectangular sample.

2. Compliance method to detect fracture/failure

Figure S2a shows the pressure (P) vs central deflection (w_0) of a rectangular plane strain membrane of half width a (400 μm) and thickness t (1.2 μm) subjected to a linearly increasing pressure from 0 to 10 kPa. The relation between pressure and central deflection of a freestanding film in a blister test is given as,

$$P = C_1 \frac{\sigma_R t}{a^2} w_0 + C_2 \frac{Et}{(1-\nu)a^4} w_0^3. \quad \dots (1)$$

The geometric parameters C_1 and C_2 are 2 and 4/3 respectively [1], for a rectangular membrane. E and ν are the Youngs Modulus and Poisson's ratio of the film, respectively. If we assume no residual stress, i.e., $\sigma_R = 0$ (which is the case for electrolyte soaked and lithiated samples) and no crack propagation, i.e., a is constant, then the response of the freestanding film is given as $P \propto w_0^3$ which is shown as blue solid curve in Fig. S2a. However, if we assume that a crack initiates and propagates steadily, i.e., a increases steadily (at, $P = P_c$, here 4 kPa in the figure), the corresponding deflection is shown as dashed black curve in Fig. S2a.

The derivative of P with respect to central deflection, i.e., dw_0/dP of eq. (1) is obtained as

$$\frac{dP}{dw_0} = \frac{3C_2Et}{(1-\nu)a^4} w_0^2 \quad \dots (2)$$

Now substituting $w_0$from eq. (1) with the assumption $\sigma_R = 0$

$$\frac{dP}{dw_0} = 3P^{\frac{2}{3}}\left(\frac{1}{C_2 Et(1-\nu)a^4}\right)^{\frac{1}{3}}, \qquad \ldots (3)$$

or

$$\frac{dw_0}{dP} = \frac{1}{3}P^{-\frac{2}{3}}(C_2 Et(1-\nu)a^4)^{\frac{1}{3}}. \qquad \ldots (4)$$

Figure S2b shows the sample compliance, i.e., dw_0/dP (eq. 4), as a function of pressure for two scenarios: 1) when the freestanding film intact (i.e., blue curve) and 2) when a crack initiated at 4 kPa and propagates. It can be observed that when a remains constant the sample compliance asymptotes following the relation $dw_0/dP \propto 1/P^{2/3}$, but when a crack initiates and propagates (e.g., at, $P = P_c$, here 4 kPa in Fig. S2) the sample compliance increases. This increase in sample compliance can be used as a method to identify or detect fracture along with visual identification.

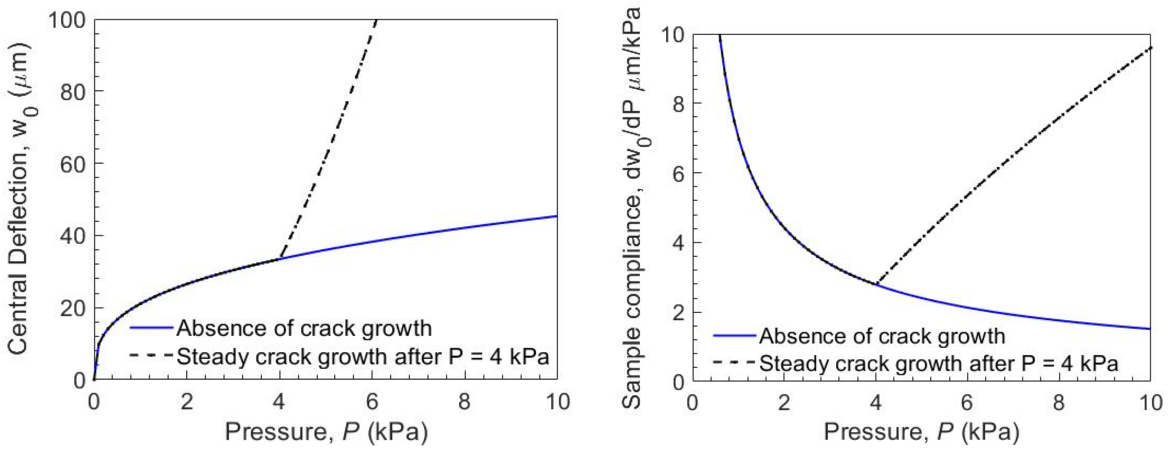

(a) (b)

Figure S2 (a) and (b) show the deflection and compliance, respectively, as a function of pressure for two different cases: i) an intact film represented by solid blue curve and ii) a film that

undergoes delamination at a critical pressure with dotted black curve. The steady increase in sample compliance (e.g., at 4 kPa in this case) is an indication of crack growth and was used to detect fracture in the samples.

3. Stress as function of distance away from crack tip

Figure S3 shows the Von Mieses equivalent stress ($\bar{\sigma}$) as a function of the distance radially away from the crack tip (along the interface) under the crack initiation pressure P_c for a PVdF/Si interface sample. Note that the stress $\bar{\sigma}$ asymptotes towards 0 MPa within 100 μm radial distance from the crack tip, and similar results were observed for PVdF(wet)/Si and PVdF(wet)/Lithiated Si samples. Therefore, as long as one includes a 100 μm sample width in the FE model, the crack tip conditions remain similar to the actual samples. In this study a substrate width of 500 μm was chosen to simulate the experimental conditions.

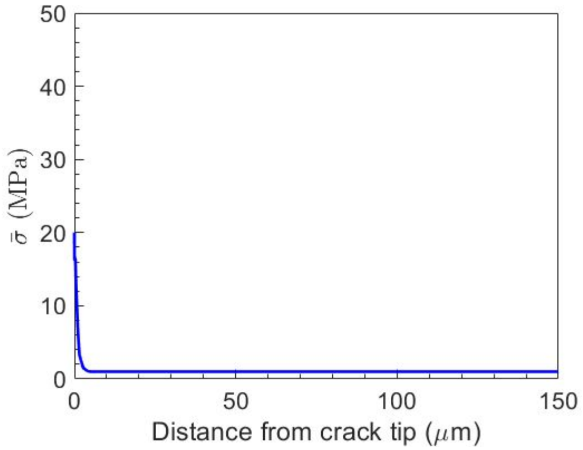

Figure S3 The von Mises equivalent stress ($\bar{\sigma}$) as function of distance from the crack tip at the PVdF/Si interface.

4. **Mechanical and fracture behavior of circular PVdF/Si interface under dry, electrolyte soaked, and electrochemical cycling conditions**

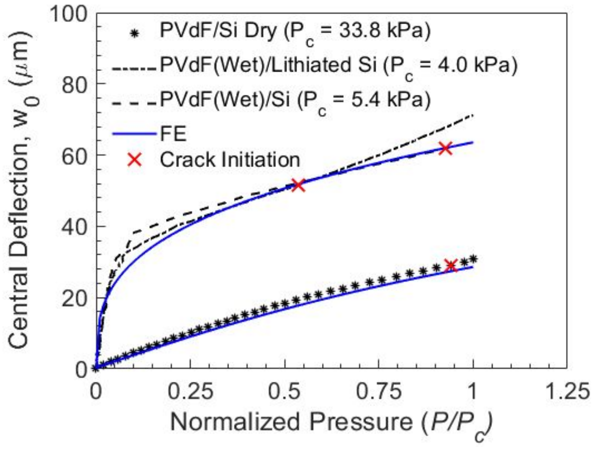

Figure S4 The central deflection vs. normalized pressure response of PVdF film of dry or as prepared sample (asterisk), PVdF(wet)/Si (dashed line), and PVdF(wet)/lithiated Si samples (dash dotted line) along with the data from the corresponding FE simulations (solid blue line) is shown. Here all samples are circular geometry.

Figures S4 shows the central deflection-pressure response of circular PVdF film in dry PVdF/Si (asterisk), PVdF(wet)/Si (dashed line), and PVdF(wet)/Lithiated Si (dash dotted line) samples during the fracture tests. Similar to rectangular samples in Fig. 7a the dry PVdF film shows a stiffer response, i.e., lower deflection for a given normalized pressure, because it not only has a higher modulus (1.6 GPa dry compared to 0.2 GPa [2–5] under wet condition) but also has higher thickness (i.e., 6 μm compared to 1.2 μm in other two samples). A good match between the experimental data and the FE

results suggests that the prescribed boundary conditions along with the material data used in the simulations are accurate. Also, to the fact that appropriate initial conditions. i.e., the residual stress and the initial profile of the free-standing PVdF film, were included in FE simulations.

5. Measurement of Initial Profile of PVdF film

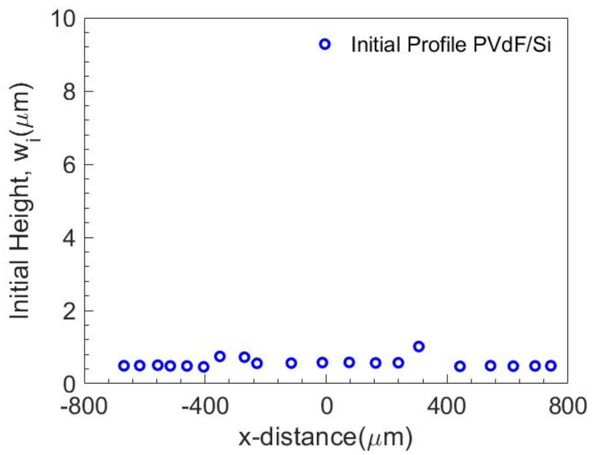

Figure S5 The initial profile of rectangular PVdF/Si dry sample measured using Zygo NewView 5000. The same is observed for all the samples including circular geometries.

6. XPS analysis of Surfaces

Table S1 Composition of various element on bare surface and fracture surface.

Surface/Composition (at%)	O	C	F	Si	N	P	Li
Pure Si surface	17.05	23.97	1.31	57.90	0.58	-	-
Pure PVdF surface	4.56	59.25	35.32	-	0.84	-	-

Dry PVdF/Si fracture Surface	35.00	19.80	1.05	42.82	1.34	-	-
Lithiated fracture Si surface	15.60	29.59	9.31	0.69	-	6.58	38.22

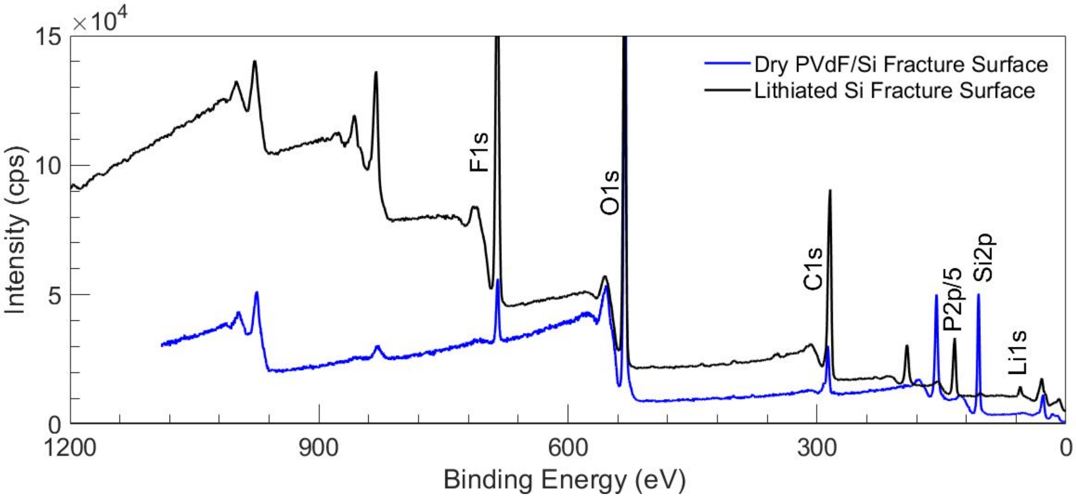

(a)

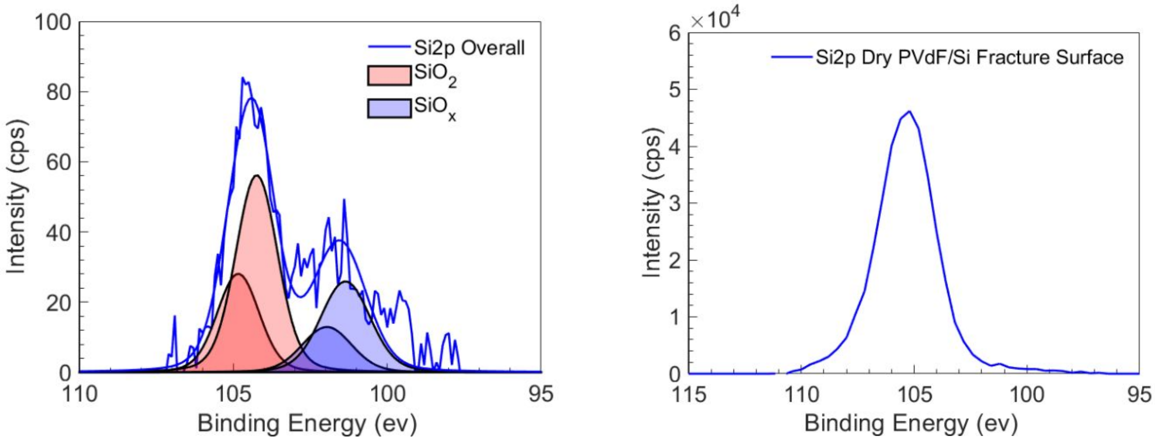

(b)

(c)

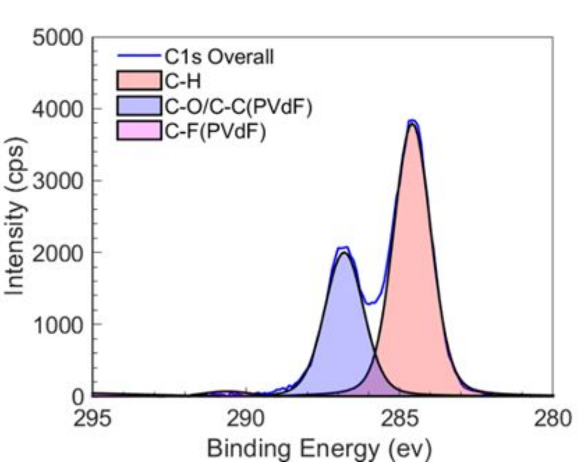

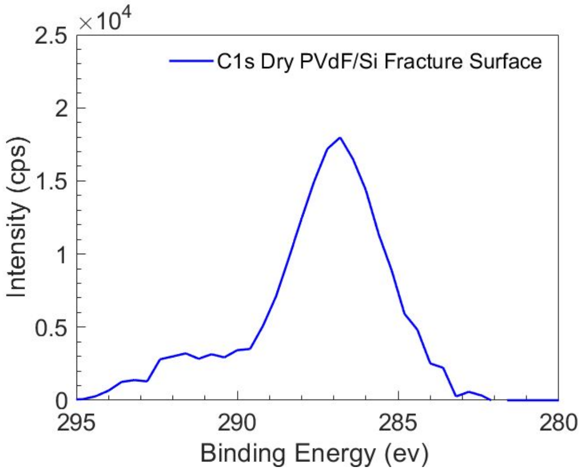

(d)

(e)

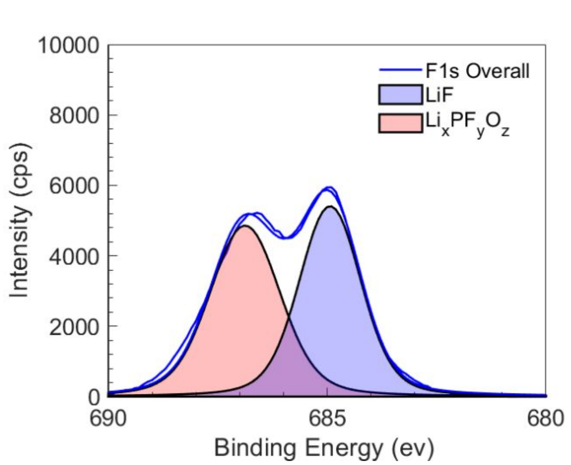

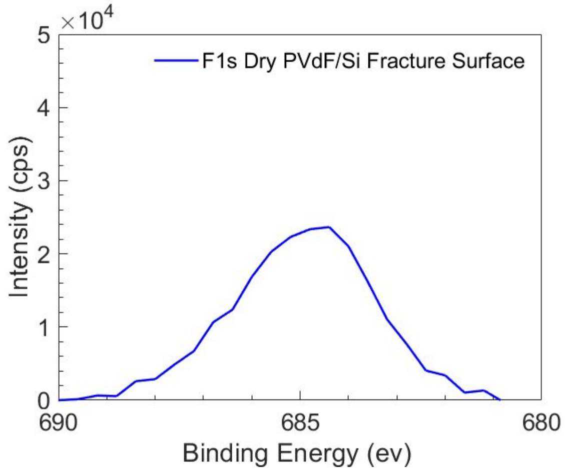

(f)

(g)

Figure S6 XPS scan (a) comparing the survey spectra and (b-c) Si, (d-e) C, and (f-g) F comparing the Lithiated Si multiplex to the Dry PVdF/Si fracture surface. The XPS analysis on both cases was conducted on Si surface.

Table S1 shows the surface element distribution on Pure Si, on the PVdF film, on the Si side of the dry PVdF/Si and lithiated PVdF/Si surfaces, respectively. Observing F composition on dry PVdF/Si and comparing that to PVdF film shows that the fracture in the dry sample occurred at the interface. This is also shown by our G_c measurement where its significantly lower that fracture of pure PVdF or pure Si. The chemical comparison between Lithiated Si surface and dry PVdF/Si can be visualized in Fig S6a which is the comparison of Si2p. Here we can observe first the lower intensity signature and peak shift and changes. If Fig. S6d-e, we similar compare C1s and Fig. S6f-g compare the F1s.   The peak shifts and intensity change in indicate the distinct chemical changes on Si surface which correlated to the drop in $G_{c.}$

References

(1) Xiang, Y.; Chen, X.; Vlassak, J. J. Plane-Strain Bulge Test for Thin Films. *J. Mater. Res.* **2005**, *20* (9), 2360–2370. https://doi.org/10.1557/jmr.2005.0313.

(2) Chen, Z.; Christensen, L.; Dahn, J. R. Comparison of PVDF and PVDF-TFE-P as Binders for Electrode Materials Showing Large Volume Changes in Lithium-Ion Batteries. *J. Electrochem. Soc.* **2003**, *150* (8), A1073. https://doi.org/10.1149/1.1586922.

(3) Magasinski, A.; Zdyrko, B.; Kovalenko, I.; Hertzberg, B.; Burtovyy, R.; Huebner, C. F.; Fuller, T. F.; Luzinov, I.; Yushin, G. Toward Efficient Binders for Li-Ion Battery Si-Based Anodes: Polyacrylic Acid. *ACS Appl. Mater. Interfaces* **2010**, *2* (11), 3004–3010. https://doi.org/10.1021/am100871y.

(4) Kovalenko, I.; Zdyrko, B.; Magasinski, A.; Hertzberg, B.; Milicev, Z.; Burtovyy, R.; Luzinov, I.; Yushin, G. A Major Constituent of Brown Algae for Use in High-Capacity Li-Ion Batteries. *Science* **2011**, *334* (6052), 75–79. https://doi.org/10.1126/science.1209150.

(5) Borges, M.; Pakhare, A.; Nadimpalli, S. P. V. In Situ Measurement of Solvent Diffusion and the Associated Changes in the Mechanical Properties of Polymer Binders. In *243rd ECS Meeting with the 18th International Symposium on Solid Oxide Fuel Cells (SOFC-XVIII)*; ECS, 2023.
